# A new subspecies of sea snake, *Hydrophis platurus xanthos*, from Golfo Dulce, Costa Rica

**DOI:** 10.3897/zookeys.686.12682

**Published:** 2017-07-24

**Authors:** Brooke L. Bessesen, Gary J. Galbreath

**Affiliations:** 1 Joyce Corrigan Memorial Care Center, 455 N. Galvin Parkway, Phoenix Zoo, Phoenix, AZ, USA; 2 Biological Sciences, 2205 Tech Drive, Northwestern University, Evanston, IL, USA; 3 Field Museum of Natural History, 1400 S. Lake Shore Drive, Chicago, IL, USA

**Keywords:** Golfo Dulce, Costa Rica, sea snake, yellow color morph, *
platurus*, type specimens, taxonomy

## Abstract

We describe a distinctive new subspecies of sea snake from the occasionally anoxic inner-basin waters of Golfo Dulce, Costa Rica, based on combined data garnered between 2010 and 2017 for 154 specimens, 123 free-ranging and 31 museum-held. The yellow sea snake, *Hydrophis platurus xanthos* Bessesen & Galbreath, **subsp. n.**, is diagnosed by a notably smaller body size and nearly uniform yellow coloration, which contrasts with the black and yellow striae and tail spots or bands typical of the species. Within the modest geographic range (circa 320 km^2^), nearly all specimens possess both diagnostic character states. Bathymetrics appear to restrict genetic flow between this allopatric population and conspecifics in the broader Eastern Pacific. In perspicuous contrast to typical *H. platurus*, *H. p. xanthos* shows no association with drift lines, and feeds at night in turbulent waters, assuming a sinusoidal ambush posture never previously reported for the species. This evolutionarily significant unit (ESU) warrants taxonomic recognition and active protection.

## Introduction

*Hydrophis platurus* (Linnaeus, 1766; as *Anguis platura*) is an elapid sea snake widespread in the Indo-Pacific region. The species was long classified in the monotypic genus *Pelamis*, but given molecular studies on its phylogenetic relationships (e.g. Lukoschek and Keogh 2006), it is most appropriately placed in the large genus *Hydrophis* (Sanders et al. 2013). At the ocean surface, this venomous piscivore is often associated with smooth-water drift lines (Kropach 1971a, 1975), where it opportunistically feeds on a variety of small fish from an open floating posture. Considered diurnal, it does not appear to spend time at the sea surface at night (Rubinoff et al. 1986; see our contrasting data below), preferring high light levels, which may suggest some reliance on visual cues for predation (Brischoux and Lillywhite 2011). The species has generally been found to actively avoid turbulent waters (Dunson and Ehlert 1971, Rubinoff et al. 1986, Cook and Brischoux 2014). Like many snakes, *Hydrophis platurus* is sexually dimorphic in size, with females larger on average. Individuals can reach at least 113 cm in total length (TL; Pickwell and Culotta 1980). Published population averages for adult TL include 70 cm (Cogger 1975), perhaps 60 cm (roughly extrapolated from Fig. 2A of Pfaller et al. 2012), and 72 cm for males/ 80 cm for females (Leviton et al. 2006). Weight averages in ecological studies have included 91 g (Graham et al. 1971) and 140 g (Rubinoff et al. 1986).

*Hydrophis platurus* is the only sea snake found off Costa Rican shores (Solórzano 2004). While variable, the majority of individuals exhibit black dorsal coloration contrasting with a yellow ventrum, and have dark spots or bands on the paddle-shaped tail (Bolaños et at. 1974; Tu 1976). Smith’s (1926) seven recognized color forms of the species did not include the yellow variety and Kropach (1971b) was the first to describe yellow individuals along the Central American coast. Later, Solórzano (2011) and Bessesen (2012) reported a population in the northern waters of Golfo Dulce to be exclusively composed of xanthic (all-yellow or primarily yellow) sea snakes. A bi-seasonal distributional study across all areas of the Golfo Dulce revealed a geographically bound aquatic habitat (circa 320 km^2^) in which 100% of sea snakes (N = 68) possessed the diagnostic character state. See Supplementary file 1. The findings were corroborated in interviews with 82 fishermen and tour boat guides, who reported seeing only yellow sea snakes in the upper Gulf. GIS maps show the xanthic population to be spatially separated from the oceanic yellow-bellied population by a gap of about 22 km (Bessesen 2012, 2015, Fig. 1).

**Figure 1.**
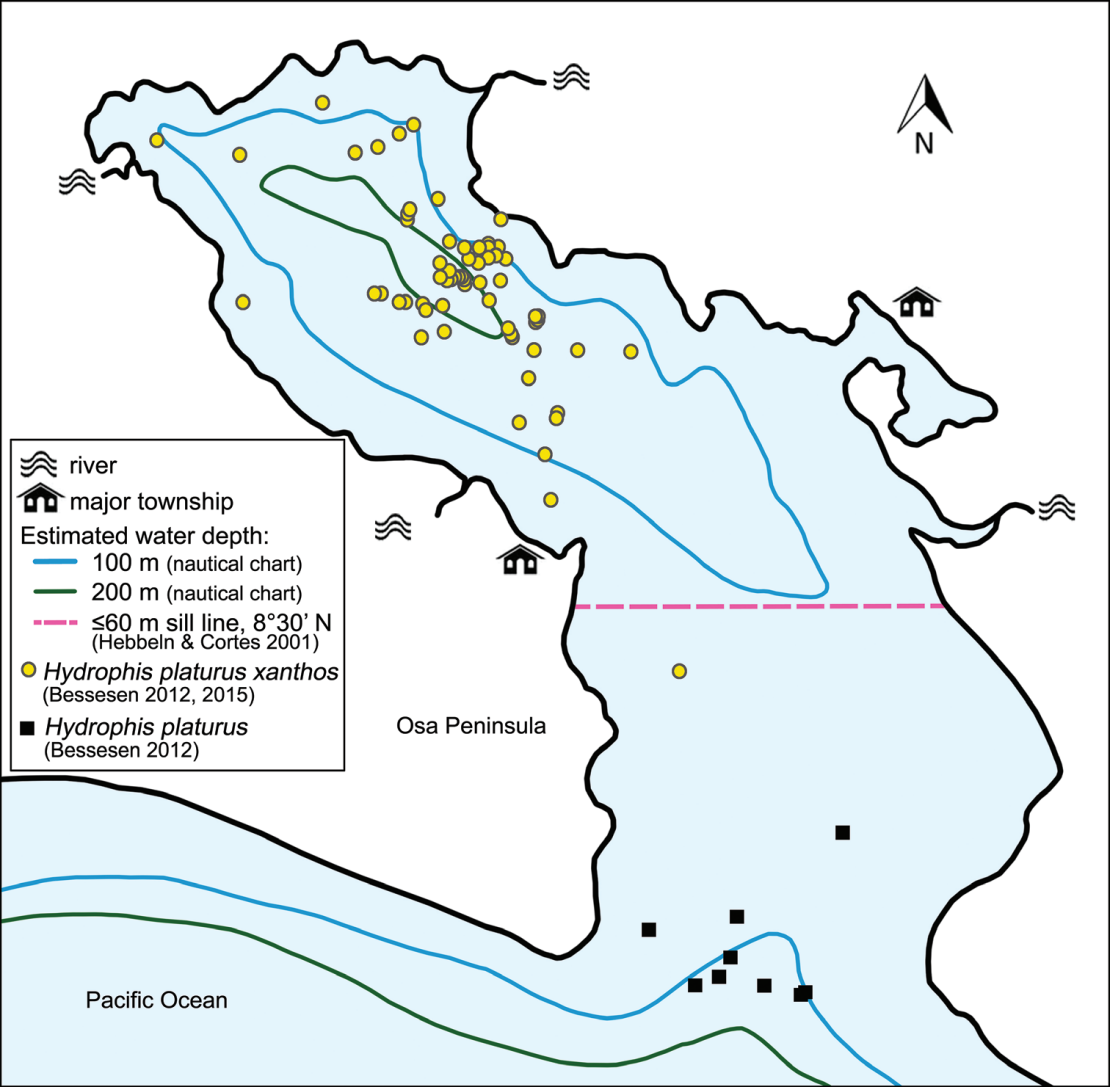
GPS sighting points for all sea snakes observed in Golfo Dulce during the 2010 and 2011 distribution study show *Hydrophis platurus xanthos* to reside in the inner basin, geographically divided from the broader Eastern Pacific population (Bessesen 2012, 2015).

This distinct population of yellow sea snakes exploits warm, periodically anoxic inner-basin waters of a curved tropical embayment with tectonic origins (Hebbeln and Cortés 2001) and fjord-like features (Wolff et al. 1996). Golfo Dulce, located on the South Pacific coastline of Costa Rica, is approximately 50 km long and 10-15 km wide, and supplied with fresh water drainage from several rivers and tributaries. The bathymetry is characterized by a 215 m deep inner basin, which is held by an effective 60 m sill and a shallow valley, ≤ 80 m deep, extending southward to the mouth of the Gulf. Such topography prevents free exchange between the deeper waters of the inner basin and adjacent coastal water masses (Svendsen et al. 2006). Graham (1974) showed *H. platurus* body temperature remained at or slightly above surrounding water, and found the serpent avoided surfacing when water temperatures were elevated (Graham et al. 1971). Since Dunson and Ehlert (1971) reported the species’ upper lethal temperature at 33°C, and sea surface temperatures in Golfo Dulce can reach 32.5°C (Bessesen 2012), higher than other areas within the range of *H. platurus* (Hecht et al. 1974), light dorsal coloring plausibly plays a role in thermal regulation (Solórzano 2011, Bessesen 2012). Bessesen (2012) and Lillywhite et al. (2015) observed behavioral differences in the xanthic population that appeared to have adaptive significance. Yellow sea snakes show no association with drift lines, are commonly seen in turbulent water, and most frequently surface at night, when they commonly assume a sinusoidal posture. The function of that sinusoidal posture was recently illuminated, as was smaller body size, during an investigation of morphology and behavior of the yellow sea snakes in Golfo Dulce, Costa Rica. The data from that study are reported here.

## Material and methods

In January 2017, 55 free-ranging yellow sea snakes were documented in the inner-basin region of Golfo Dulce. Time and Beaufort Wind Force (BWF) were recorded at each sighting, as was the behavior of the snake: resting (floating at the surface), swimming, knotting, feeding, breeding and/or avoidance (diving). The posture of each snake was also recorded; open (elongated) versus sinusoidal. Forty-three of the specimens were captured by dip net for collection of morphological data, including total length (TL), tail length, paddle height, weight and high‐resolution photos of key physical characteristics. Each snake was given an effective calming mask using 1-inch Vetrap wrapped around the head, and allowed to quiet. While resting still, TL was obtained by laying string along the mid-dorsal surface from tip of snout to tip of tail, and then measuring the string against a meter stick, taking just two measurements if the results matched exactly, or if not, averaging three measurements diverging by no more than 0.5 cm. Tail length was measured against a meter stick to nearest 0.5 cm. Paddle height was measured in 34 specimens with calipers to 0.5 mm. Weight was measured on an AWS Blade-1KG digital scale; if boat movement created a swing in readout, care was taken to wait until a proper estimate could be acquired. An additional behavioral note was made if a snake gaped its mouth or attempted to bite during handling. On-water work periods began no later than 16:30, lasting an average of 6 hr, 10 min. Sea surface temperature was measured with a floating water thermometer once per evening.

Following field work, 31 preserved specimens of *Hydrophis platurus* were examined at the Zoological Museum of University of Costa Rica; all had been captured in the upper Golfo Dulce between 2009 and 2016, and all exhibited xanthic coloration. Five particularly small specimens (<38 cm TL) were excluded from measurement to avoid possible age-related statistical bias; however, morphological data were collected for the remaining 26 specimens using the same techniques as applied in the field. TL was measured by string, although merely one precise effort was required. Tail length, paddle height, weight and photos were also collected. No additional morphological counts, such as scalation, could be practicably obtained owing to limited access to the preserved specimens.

During the research period, opportunity also arose to measure three adult yellow-bellied specimens, two preserved and one living, found along the Osa Peninsula. The same tools and techniques were applied.

## Results

**Figure 2.**
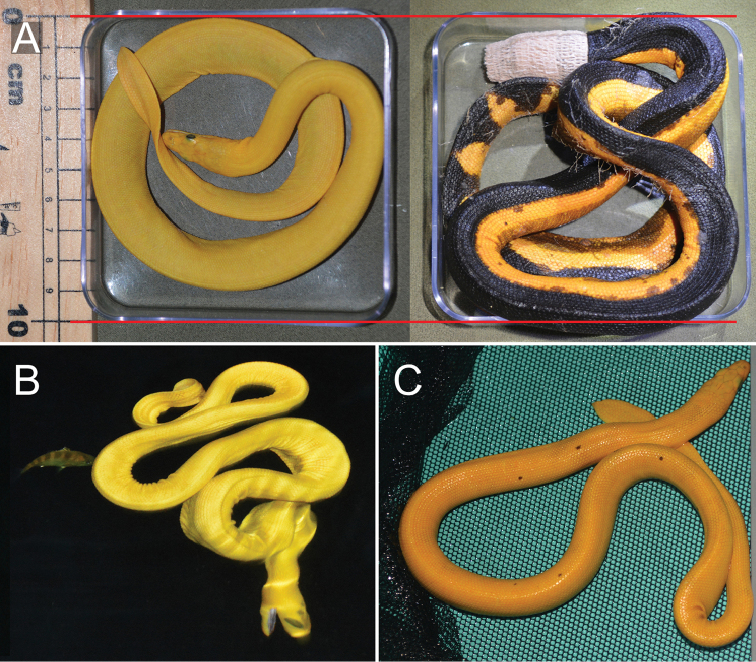
*Hydrophis platurus xanthos* sharply contrasts typical *H. platurus* in color, body size and behavior. **A** Scaled size comparison of yellow sea snake, TL 43 cm (left), and yellow-bellied sea snake, TL 69 cm (right; note the use of Vetrap as a calming mask and sickly condition of the specimen) **B** ambush posture of *H. p. xanthos*; floating at the sea surface at night in a sinusoidal shape, head below, mouth agape **C** although predominantly yellow, xanthic individuals often possess black spots along the dorsum.

A total of 69 yellow sea snakes collected from the inner basin of Golfo Dulce was measured and weighed. TL ranged from 40–59 cm, with a mean of 49.1 cm. Tail length ranged from 4.3–7 cm and averaged 5.4 cm, significantly shorter than the typical 8–9 cm described by Leviton et al. (2003); Cook and Brischoux (2014) estimated mean proportionate tail length at 11.2% of TL, which held true for our samples. Weight ranged from 22–95 gm, with a mean of 46.6 g (Table 1). In comparison, the three measured yellow-bellied specimens had a TL ranging from 69–70.5 cm, with a weight average of 95 g; such findings are in accord with published size descriptions for *H. platurus* (Fig. 2A). While comparative data for paddle height is limited, we found xanthic specimens to have an average height of 9.8 mm, while the yellow-bellied specimens averaged 11.8 mm.

**Table 1.** Body measurements for 69 individual *Hydrophis platurus xanthos* from the inner basin of Golfo Dulce.

Specimen	TL (cm)	Tail length (cm)	Paddle height (mm)	Weight (g)
F1	49	5.5	-	28
F2	46	5.5	-	22
F3	52.5	4.5	-	50
F4	50	5	-	47
F5	48	4.5	9	35
F6	48	4.5	9.5	30
F7	46.5	5	9.5	28
F8	52	5	9	44
F9	48	4.5	9	29
F10	47	4.5	9.5	37
F11	50.5	5	9.5	42
F12	55.8	6	9.5	38
F13	47	4.5	-	32
F14	48.5	5.5	8	33
F15	47.6	4.5	9.5	39
F16	46.8	4.5	9	33
F17	55	5.5	10	52
F18	48.3	4.5	10	43
F19	46	4.5	9.5	38
F20	46	4.5	-	35
F21	49	6	9	34
F22	45.5	5.5	-	37
F23	50	5	10	52
F24	47.8	5	9.5	46
F25	45.8	5	9.5	32
F26	51.5	6	10	53
F27	45.2	5	9.5	32
F28	46.8	5	9	34
F29	48.7	5.5	9.5	41
F30	46.5	5.5	9	32
F31	54.3	6	10.5	61
F32	49.8	5.5	9.5	42
F33	49.5	6	-	38
F34	50.5	6	-	48
F35	48	5.5	8.5	40
F36	41	5	8.5	26
F37	45.3	5	9	40
F38	46	5.5	9.5	38
F39	47.7	5.5	11.5	45
F40	44	5	9.5	37
F41	48.3	5.5	9	43
F42	43	4.5	9.5	32
F43	50.8	6	10.5	48
M1	57.5	6.5	10.5	59
M2**	52	6	10	55
M3	59	7	11	72
M4	56	6	14	95
M5	51.5	6.5	14	73
M6	50	6.5	12	71
M7	51	6	9.5	54
M8	51	6	10	73
M9	51.5	6.5	10.5	94
M10	45	5.5	9	35
M11*	51	6	10	55
M12	42	4.5	9	26
M13	52	5	9.5	65
M14	55	6	11	78
M15	51	6	10	74
M16	55	5.5	11	94
M17	45.5	6	9.5	38
M18	54.5	5.5	10.5	80
M19	40	4.5	9.5	35
M20	50.5	5	9.5	79
M21	54	6	10	46
M22	46	5	8	35
M23	44.5	5	10	38
M24	50.5	6	9.5	40
M25	48	5	10	47
M26	50	4.5	8	36
Mean:	49.1	5.4	9.8	46.6

Notes: F specimens were free-ranging, captured during field studies, while M specimens were examined at the Zoological Museum of University of Costa Rica; **holotype; *paratype.

Several free-ranging yellow sea snakes were seen swimming (N = 13) and a few knotting (N = 3), but the most commonly observed surface behavior was resting. Recorded evening sea surface temperatures in the inner basin averaged 28.5°C, considerably cooler than the average 30.5°C previously recorded near the Puerto Jiménez marina in the dry season of 2010 (Bessesen 2012). BWF was documented at every sighting to provide specific insight into turbulence associated with xanthic snakes at the sea surface. Of the 55 live yellow sea snakes recorded, none were seen in glassy or rippled water. Only one was found in BWF2 (small wavelets), whereas the majority were recorded in BWF3 (N = 33; smooth wavelets) or BWF4 (N = 21; small wavecaps). On two nights when sea conditions calmed from BWF3 to BWF2, snakes could no longer be located. This association with rough water is in direct contrast to the behavior of typical yellow-bellied sea snakes, which strongly favor calm smooth water for surfacing. Furthermore, data reconfirmed that yellow sea snakes most commonly surface at night, at which time they often assume a tight sinusoidal posture that has never been observed during daylight hours (Fig. 2A). The first yellow sea snake sighted post-sunset was always sinusoidal, a posture observed in 80% of nocturnal sightings. Perhaps the most unexpected finding was that the sinusoidal body position appears to represent the typical ambush posture of yellow sea snakes (Fig. 2B). Six yellow sea snakes were observed in this feeding position with small prey around them, and one was captured with a larval fish in its mouth. Bunching up the body appears to create a stable buoy in turbulent water conditions while the head extends below, mouth agape. In hand, the yellow sea snakes were not overly aggressive; of the 43 brought aboard our research panga for examination, only 33% (N = 14) gaped their mouth at any point. Avoidance behavior prevented us from measuring 12 living specimens, which were seen and recorded, but not caught, before diving.

During the 2017 field work, three yellow-bellied *H. platurus* were unexpectedly found in the inner basin of Golfo Dulce. All were within close proximity (two within 400 m and another 2 km to the south). Two were juveniles, weighing only 12–22 grams. The third was an adult, which appeared to be dying, limp in the water with head drooping. Its body was emaciated, with ribs palpable, and slimy grey plaques covered the head and speckled the skin. Strings reminiscent of thread algae also protruded along its length (Fig. 2A). Local fishermen were flummoxed by news of our finding and reiterated their experience, as reported in the 2010–2011 study: None had ever seen a yellow-bellied snake in upper Golfo Dulce.

### Description of a new taxon

#### Hydrophis platurus xanthos subsp. n.

Taxon classificationAnimaliaSquamataElapidae

http://zoobank.org/291A79DC-C871-4AE3-9628-A60AE9CF9445

##### Type specimens.

Holotype female from COSTA RICA: Golfo Dulce: inner basin, 08°35.76'N; 083°13.25'W; collected 13 February 2009 by A. Solórzano, and deposited in the Zoological Museum of University of Costa Rica, San Jose, Costa Rica (MZUCR:HERP:20614); body yellow with 4 black specks (<1 mm) and 5 black dots (2–4 mm) along the dorsum, no tail markings; TL 52 cm, tail length 6 cm, paddle height 11 mm, weight 55 g. Paratype female from COSTA RICA: Golfo Dulce: inner basin, 08°35.76'N; 083°13.25'W; collected 2 September 2009 by A. Solórzano, and deposited in the Zoological Museum of University of Costa Rica, San Jose, Costa Rica (MZUCR:HERP:20836); body yellow with 4 dark-brown dorsal blotches (2–3 mm) on or just caudal to the head, 2 black specks (< 1 mm) and 2 dots (3–4 mm) farther down the dorsum, no tail markings; TL 51 cm, tail length 6 cm, paddle height 10 mm, weight 55 cm.

##### Diagnosis.

Here we describe a new, allopatric subspecies, *Hydrophis platurus xanthos* subsp. n., or yellow sea snake, from the inner basin of Costa Rica’s Golfo Dulce. The new subspecies is diagnosed based on a dramatic color character state, as well as by a marked difference in body size. Aspects of behavior also appear to be unique.

*Hydrophis platurus xanthos* is diagnosed as differing from other *H. platurus* by its predominantly yellow coloration and smaller size. Apparent additional behavioral diagnostic traits include a sinusoidal ambush posture, and a preference for surfacing in rough rather than smooth waters, lacking an association with drift lines. No specimens unambiguously assignable to this taxon have shown full lateral striation on head and body or prominent spots or bands on the tail. The appearance of *H. p. xanthos* starkly contrasts with the coloration of most conspecifics found in the broader Eastern Pacific, or even in the adjacent mouth of the Golfo Dulce, which evince nearly solid black pigmentation along the dorsum, breaking into spots or bands at the caudal end (Fig. 2A). *H. platurus xanthos* is also shorter in length, with an average adult TL of 49 cm, compared to about 60–75 cm for other *H. platurus* populations. Comparing our TL data for *H. p. xanthos* with those of Rubinoff et al. (1986) for *H. platurus* in the Gulf of Panama, note that the two samples do not even overlap, despite good sample sizes. The difference between the underlying distributions is significant with P < .01 (Wilcoxin Rank Sum Test, N1 = 15, N2 = 69, T1 = 1155). Behavioral differences are equally pronounced between *H. platurus xanthos* and its yellow-bellied conspecifics. Drift lines, which play a critical role in the natural history of the species, supporting aggregations for feeding, reproduction and transport, are not used by *H. platurus xanthos*. Furthermore, while *H. platurus* feeds diurnally, stretched out in smooth water, *H. platurus xanthos* feeds at night, in turbulent water, assuming a sinusoidal ambush posture never previously reported for the species.

##### Coloration detail.

Head, body and tail are bright canary-yellow. Although appearing entirely yellow at a glance, most individuals possess at least one dark, black or brown, dorsal speck or dot (Fig. 2C); such markings lack any fixed pattern, but frequently present caudal-medial to the supraocular scales. A few specimens retain larger black spots along the dorsal ridge; of the yellow sea snakes photographed by an author (BLB; N = 120), less than 13% (N = 15) exhibited a black mark longer than 2 cm.

##### Etymology.

From Greek *xanthos*, “yellow,” to highlight a diagnostic feature of this subspecies.

##### Geographic distribution.

The breeding population of *Hydrophis platurus xanthos* appears confined to approximately 320 km^2^ in the northern half of the Golfo Dulce, Costa Rica, from 08°32'N to 08°44'N and 083°11'W to 083°28'W. A spatial gap up to 22 km separates the yellow sea snakes from the usually bi- or tricolored oceanic population, and appears to restrict genetic exchange (Bessesen 2012, Fig. 1).

## Discussion

*Hydrophis platurus xanthos* constitutes a geographic isolate in which most or all specimens conform in possessing the diagnostic color state, otherwise known in very few individuals of other populations of this wide-ranging species. Sheehy et al. (2012) compared mitochondrial DNA from two Costa Rica populations, the xanthic population in Golfo Dulce, and a yellow-bellied population in Golfo de Papagayo. Four statistical tests for difference were utilized. Two of them, Ks* and Z*, found significant difference at *P* < .05; two others, X^2^ and Hs*, did not. That no shared cytochrome-b haplotypes were found is intriguing, but the sample sizes were small. We concur with Sheehy et al. that any real difference in mtDNA between these populations is itself probably small and implies shallow phyletic divergence. This is not in conflict with taxonomic differentiation of the Golfo Dulce snakes, since subspecific or even specific differentiation can occur rapidly under circumstances of intense selection and/or genetic drift.

The yellow sea snake, *H. p. xanthos*, is also notably smaller in size than the yellow-bellied variety. At an adult TL of about 49 cm, weighing approximately 47 g, it measures around 10–25 cm shorter and 33–50% lighter in weight than published averages for *H. platurus*. A portion of that difference could relate to measuring technique: string versus stretching (Fitch 1987, Rivas et al. 2008). However, while typical female members of the species found in the Eastern Pacific may not commence reproductive activity until they reach a TL of 64.5–76.5 cm (Kropach 1975), no recorded individuals of the xanthic taxon even encroach into that size range. Behavioral differences suggest that the unique habitat of *H. p. xanthos* has imposed adaptive change. In Golfo Dulce, *H. platurus* has not only made a dramatic shift in tolerance—indeed, preference—for turbidity, correlated with a sinusoidal ambush posture, but appears to have shifted from diurnal to nocturnal predation. If insulated bathymetrics and restricted water currents within Golfo Dulce interrupted transit and reproductive flow with the wider oceanic population in the past, the need to avoid overheating may have selected for all-yellow coloration to reduce absorption of solar energy while surface feeding. Perhaps that same environmental pressure led to a nocturnal feeding strategy and may also explain why *H. p. xanthos* concentrate in the cooler deepest waters of the inner basin (Fig. 1).

*Hydrophis platurus* is very widely distributed, yet has remained largely geographically undifferentiated in morphology. That fact makes the singular xanthic population particularly intriguing. Several interesting questions are raised. For example, does the yellow color morph represent a small but normal percentage of the oceanic population in the Eastern Pacific? When 102 sea snakes were collected from five locations along the northern Pacific coastline of Costa Rica, only one (1%) was described as “yellow with a few black dorsal dots” (Bolaños et al. 1974). Of 3,077 specimens collected near Bahia de las Culebras, four (0.1%) were yellow (Tu 1976). Yet Kropach (1971b) found the variant in 9 of 278 specimens (3%) near the mouth of the Golfo Dulce. That xanthic specimens increase closer to our described population suggests the possibility that xanthic individuals in the Eastern Pacific have washed out from the inner basin of Golfo Dulce. To that point, all but one of the 154 yellow sea snake documented by an author (BLB) occurred well within habitat boundaries. However, a single xanthic specimen was observed in the outer basin in 2011 when rainy season storms and higher waves prevailed. This finding indicates that individuals of the subspecific colony occasionally cross the sill line, and might ultimately emigrate into the broader Pacific population (Fig. 1). More intriguing is our discovery of yellow-bellied specimens above the sill line; to our knowledge, no others have ever been sighted in the upper Gulf. While cause of disease in the emaciated, algae-coated adult found dying remains unknown, sea snakes are quite sensitive to rises in water temperatures (Gillett 2015). How did the yellow-bellied snakes come to be in the inner basin? It may be relevant that their appearance occurred about seven weeks after the severe weather event of Hurricane Otto, which caused record-breaking precipitation on the Osa Peninsula. How frequently yellow-bellied snakes may be washed into the embayment or whether they can or do produce viable progeny with resident yellow sea snakes is unknown.

How do Golfo Dulce’s periodically anoxic conditions impact the health, metabolic rate and/or behavior of the yellow sea snake? Despite being an air-breathing reptile, the species is reported to spend up to 99.9% of its time at 20–50 m depth, remaining submerged for up to 213 minutes per dive (Rubinoff et al. 1986). Below the water surface, *H. platurus* can absorb 12–33% of oxygen through skin respiration (Graham 1974). How might those physiological and behavioral characteristics be altered when the surrounding seawater lacks normal levels of dissolved oxygen? And, if *H. platurus* uses visual cues for predation, how does a nocturnal feeding schedule influence detection of prey?

An important unknown is the population size of *H. p. xanthos* in Golfo Dulce. Although the taxon is not at imminent risk of extirpation, it constitutes a very geographically limited population (inhabiting only circa 320 km^2^ of aquatic habitat). This ESU is deserving of *in situ* conservation. Whether endemic to an island, mountain, lake, or gulf, any population confined to such a narrow habitat that is affected by human activity is at risk of decline and potential extirpation. Reading et al. (2010) observed that snake populations appear in global decline, and noted that populations living in unprotected habitats with increasing human influence are at greatest general risk of collapse. Golfo Dulce is not yet a Marine Protected Area, and anthropogenic impacts are already negatively affecting the marine environment (Spongberg 2004, Quesada-Alpízar and Cortés 2006, Bessesen 2015). Since the presence of yellow sea snakes in Gulfo Dulce was first recognized, they appear to have become an attraction for visitors. In 2009, only one photo of a yellow sea snake was found online by an author (BLB); in 2016, there were multiple online sightings, Facebook posts, and YouTube videos. While fascination with *H. p. xanthos* is in some ways positive, it also potentially endangers the population. We note with concern, for instance, that collectors have reportedly begun removing xanthic specimens from Golfo Dulce. Conservation measures related to regulating collection of this newly recognized taxon may prove necessary. We add that, living at the upper edge of the species’ temperature tolerance, there is also potential for population destruction from climate change. While it is important to recognize *H. p. xanthos* taxonomically, we have concern that taxonomic recognition without protection could lead to yet greater interest from global collectors, and urge that immediate preservation measures be considered in response to our subspecific designation.

This population has experienced genetic isolation sufficiently long to transition to an essentially monochromatic organism of notably smaller than usual stature. Major coloration and size changes evinced by *H. p. xanthos* are almost surely genetic, though of course we cannot identify the nuclear genes involved, and these are phenotypic traits for which such major changes would presumably involve notable changes in absolute individual fitness. Adaptive aspects of the taxon’s behavioral ecology may be partly or wholly a result of past natural selection on behavior; however, we do not know the relative roles of selection and drift, nor the degree of phenotypic plasticity involved.

Hopefully this globally unique population can continue to offer both scientists and conservation-conscious tourists a worthy subject of observation and study.

## Conclusions

*Hydrophis platurus xanthos* is a well-defined evolutionary subspecies inhabiting a small area of unusual geography. Given unique aspects of its behavioral ecology, it could well represent an intrinsically genetically isolated taxon of recent origin, in which case a species designation would be appropriate. We have been appropriately conservative here, in defining it at a subspecific level. This provides footing for protective strategies, while allowing future research to refine its taxonomic rank.

## Supplementary Material

XML Treatment for Hydrophis platurus xanthos

## Supplementary materials

Supplementary material 1 Table of localities Data type: distribution Explanation note: *Hydrophis platurus xanthos*, COSTA RICA: Golfo Dulce: inner basin, 2010–2011. This dataset is made available under the Open Database License (http://opendatacommons.org/licenses/odbl/1.0/). The Open Database License (ODbL) is a license agreement intended to allow users to freely share, modify, and use this Dataset while maintaining this same freedom for others, provided that the original source and author(s) are credited.Brooke L. Bessesen, Gary J. Galbreath
